# Snapshot of Defense Systems in Multidrug Resistant *Klebsiella pneumoniae*

**DOI:** 10.17912/micropub.biology.001752

**Published:** 2025-08-20

**Authors:** Tosin Yetunde Senbadejo, Samuel Ntiamoah Osei, Abiola Isawumi

**Affiliations:** 1 West African Centre for Cell Biology of Infectious Pathogens, Department of Biochemistry, Cell and Molecular Biology, College of Basic and Applied Sciences, University of Ghana, P. O. Box LG54, Legon, Accra, Ghana

## Abstract

Bacterial defense mechanisms protect pathogens from host immunity, bacteriophages, and harsh environments. This study investigates defense systems in multidrug-resistant *Klebsiella pneumoniae* from Ghanaian hospital ICUs, focusing on CRISPR-Cas, restriction-modification (R-M), and toxin-antitoxin (TA) systems. Genomes of environmental (NS2) and clinical (PS4) strains were sequenced and analyzed using PADLOC, defensefinder, and TADB3.0. NS2 carries 12 defense systems, including CRISPR-Cas, while PS4 has five. Both possess diverse RM and TA systems. These strains, resistant to six antibiotic classes, encode clinically significant defense systems, suggesting microbial exchange between fomites and humans, potentially increasing infection risks in ICU environments requiring targeted surveillance.

**Figure 1. Genomic Features and Defense Systems in Klebsiella pneumoniae:**
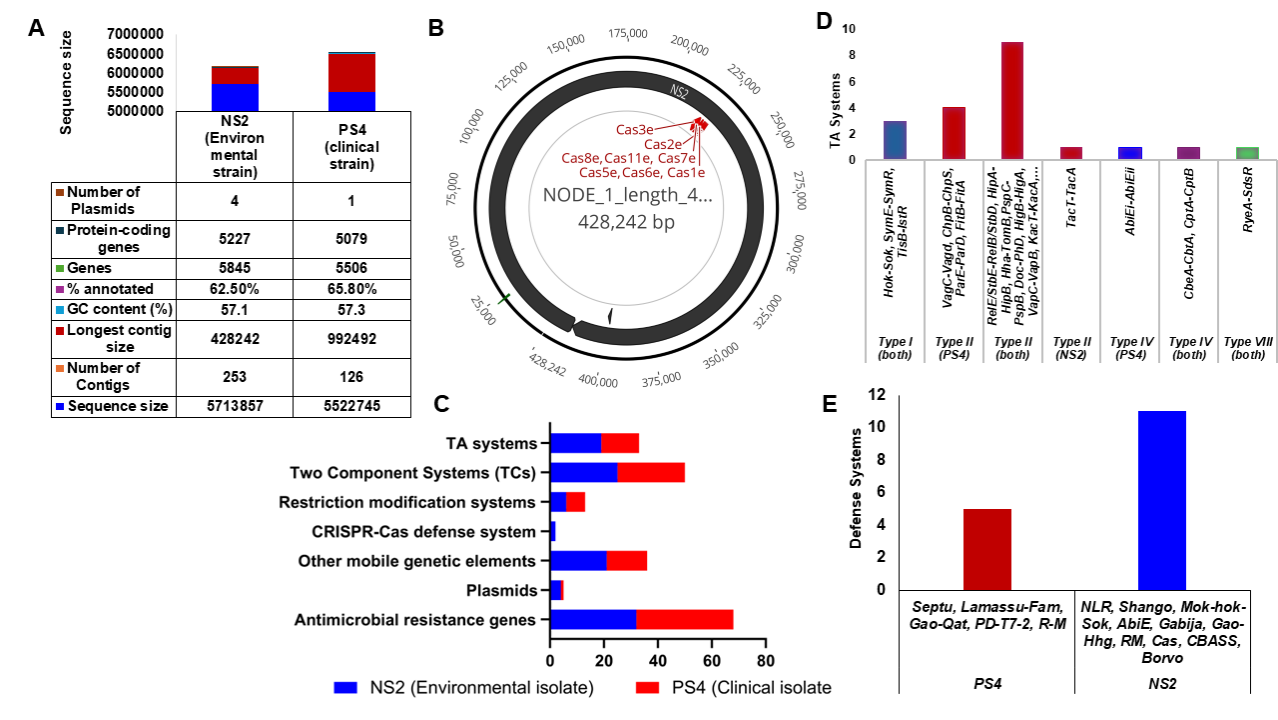
(a) Comparative genome features of *Klebsiella pneumoniae* strains. NS2, the environmental strain has a bigger genome than the PS4, the clinical strain. (b) CRISPR-Cas identified in clinical isolate (PS4). (c) Defense systems including toxin-antitoxin systems, restriction and modification systems and other features in the *Klebsiella pneumoniae* strains. (d) The shared and unique Toxin-antitoxin (TA) types in the strains (e) Other defense systems in the strains.

## Description

*Klebsiella pneumoniae* is an opportunistic pathogen implicated in hospital-acquired and community associated infections (Liang et al., 2022). As multidrug resistant pathogen, it contributes to public health challenges with consequent 22% to 72% annual mortality and morbidity rate (Poerio et al., 2022). Clinical and environmental *K. pneumoniae* shares antibiotic resistance (AMR) phenotypes, virulence and resistance genes (Rocha et al., 2022). Its severity and invasiveness is attributed to genome complexity and acquisition of factors facilitating persistence and evasion of host immune systems (de Sales et al., 2022). Some of these genomic factors are defense systems that play relevant roles in virulence and spread of multidrug resistance.

CRISPR-Cas, TAs, and restriction-modification (R-M) are defense systems involve in bacterial colonization, virulence regulation, biofilm formation, persistence, spread of genetic elements, and immunity against phages (Horesh et al., 2020). CRISPR-Cas systems in *K. pneumoniae* interfere with acquisition of phages and genetic elements that harbor AMR genes; therefore reduce AMR levels (Li et al., 2018). It has also been indicated that they can be explored for development of novel antimicrobial agents in the treatment of MDR *K. pneumoniae* infections (Xu et al., 2019).

Restriction and modification systems protect host bacterial cells from viruses and other bacteria (Bogdanova et al., 2008). R-M system is the most abundant and studied defense system in prokaryotes (Tesson et al., 2022). Type II R-M is the most abundant in bacterial genomes and in *K. pneumoniae* , KpnAI, KpnBI, Kpn2I are the most common (Chin, 2000). They provide the first line of immunity with restriction endonuclease (R) and methyltransferase (M). The R recognizes foreign DNA sequence and introduces double-stranded breaks around the recognition site while the M recognizes host DNA sequence and methylates to prevent cleavage and site recognition by R (Bogdanova et al., 2008). The type II systems are mostly plasmid-encoded for horizontal gene transfer (Rodic et al., 2017) and ensure bacterial persistence.

Toxin-antitoxin systems contribute to bacterial cell growth arrest and survival (Li et al., 2023). Also, it helps in genetic material maintenance, biofilm formation, resistance to stresses, virulence and pathogenesis (Kamruzzaman et al., 2021). TAs comprises a stable toxin and a labile antitoxin that can either be protein or RNA depending on the type. There are eight chromosomal or plasmid-mediated TAs (Type I - VIII) observed in bacterial genomes (Jurėnas et al., 2022). In Ghana, there is a paucity of data on defense systems in MDR *K. pneumoniae* , especially those isolated from Intensive Care Unit (ICU). It is therefore important to profile defense systems in the newly emerging MDR clinical and environmental *K. pneumoniae* , as AMR is increasingly becoming a burden in Ghana.

**General Features and defense systems of multidrug resistant *Klebsiella pneumoniae***

Based on the genome sequencing, NS2 has 5,713,857 read sequences in 253 contigs with a GC content (57.1%), 5751 coding sequences and 94 RNAs while PS4 has 5,522,745 sequences in 126 contigs with a GC content (57.3%), 5413 coding sequences and 93 RNAs. Only 62.5% and 65.8% of the strains could be annotated with RAST and PROKKA v1.14.6 ( **Figure 1A** ). The strains have similar features with annotated reference *K. pneumoniae* . They have ANI values of 98 – 99% with those from database based on BLAST and MUMmer results. The genomes of the strains contain chromosome and plasmid-borne Type I, II and IV R-M systems. They are also abundant in the ‘pathogenicity islands’ indicating possible roles in pathogenicity and virulence. CRISPR-Cas systems including CRISPR-Cas Type I and subtype I-E were found in NS2 environmental isolate ( **Figure 1B** ). In similar study, Type I-E and subtype I-E were dominant in clinical isolates in association with higher virulence, acquisition of plasmids, other MGEs and MDR genes in *K. pneumoniae* (Alkompoz et al., 2023). NS2 as an environmental strain has a larger genome, more plasmids, hypothetical proteins and other mobile genetic elements including transposons and insertion sequences than PS4 ( **Figure 1C** ). Plasmids harbor genes that encode resistance and virulence. It has been indicated that *K. pneumoniae* resident on fomites (medical devices) harbor resistance markers, plasmids and stress-related genes (Rocha et al., 2022). The clinical isolate (PS4) carried MGEs encoding genes that could confer AMR, heavy metals tolerance and stress adaptation. In comparison with reference strain, NS2 and PS4 have 36 genes (14 systems) and 17 genes (6 systems) respectively. This might indicate the acquisition of extra defense systems from the environment or through selection pressure, especially at the ICU with significant reliance on antibiotics. CRISPR-Cas and R-M systems contribute to evolution and interactions between MGEs and bacterial hosts (Koonin & Makarova, 2017). There are more than thirty characterized and novel TAs. Three of the four NS2 plasmids have TAs (Supplementary). The frequency of Type II TAs are higher ( **Figure 1D** ), similar to previous study (Sberro et al., 2013). Majority of the TAs were localized in the chromosomes, contrary to what has been reported (Leplae et al., 2011). Also, some of the TAs were in the genomic island alongside R-M systems and other virulence genes.

To investigate potential horizontal gene transfer (HGT) between NS2 and PS4, both genomes were screened for mobile genetic elements and pairwise whole-genome alignments using stringent homology criteria of ≥95% nucleotide identity and ≥80% sequence coverage. Pairwise whole-genome alignment (dnadiff/nucmer; delta-filter -1) between NS2 and PS4 produced 269 collinear alignment blocks, collectively covering 4,993,204 bp of sequence, and identified 35,426 SNPs across aligned regions (~7.10 SNPs per kb). We then compared the plasmid and mobile element content of NS2 and PS4 to search for evidence of HGT. NS2 carries four plasmids and 12 additional MGEs, while PS4 carries one plasmid and 27 MGEs (transposons/IS). Despite genome-wide divergence, there were 12 MGEs that are highly conserved between the two strains, with percentage identities ranging from 97.3% to 100% and sequence coverages from 80% to 100%. The conserved MGEs include both insertion sequences (e.g., IS6100, IS5075, ISEc9, ISKpn12) and larger elements such as integrative conjugative elements (e.g., ICEEcoED1a-1). The high sequence identity and large alignment length indicate horizontal transfer events between the two strains. Comparing the predictions from PADLOC, DefenseFinder, and TADB 3.0 across all genomes, the discrepancies were minor and inconsequential. The tools produced consistent results in identifying the major defense systems, with greater than 90% agreement in system classification. For example, AbiEi/AbiEii was predicted by all three tools, whereas hok–sok was detected only by DefenseFinder and TADB. Minor discrepancies were observed, particularly in system subtyping and partial system predictions. For instance, DefenseFinder identified Gao systems absent from PADLOC results, likely due to differences in underlying HMM profiles and classification thresholds. TADB 3.0, which is specific for toxin–antitoxin systems, detected additional TA loci not reported by the other tools, likely because PADLOC and DefenseFinder focus on defense islands. These differences reflect variation in content of the database, detection thresholds, and annotation criteria, and these were considered in our interpretation of results because only unique genes and systems were selected from all tools.

**Conclusion**

This is the first study to characterize CRISPR-Cas, R-M and TAs in environmental and clinical *K. pneumoniae* strains from Ghanaian hospital ICUs. The strains have clinically relevant and comparable defense systems, thus indicating possibility of fomites-humans’ microbial exchange. This can also promote the spread of AMR, virulence genes and increase the risk of hospital acquired infections. The high levels of TAs and R-M in the genome of the strains might increase the expression of MGEs, AMR virulence genes and facilitate bacterial persistence under stress. These defense systems could serve as tools for prevention and control of MDR *K. pneumoniae* considering their abundance and contribution to virulence and pathogenicity.

**Materials and Methods**

**Isolation, characterization and annotation of the bacterial strains**

*Klebsiella pneumoniae* (NS2 and PS4) strains were obtained from ABISA Bacterial Culture Library at West African Centre of Cell Biology of Infectious Pathogens, University of Ghana. They were isolated from septicemia patients (PS4) and hospital ICU fomites (NS2). The gDNA was extracted using QIAGEN bacterial-DNA kit, genome sequenced on Illumina platform following library preparation. Rapid Annotation Subsystem Technology (RAST) was used for gene prediction and annotation of assembled sequences. Average Nucleotide Identity (ANI) was determined with JSpecies-WS. The nucleotide and protein sequences of the coding genes were used as data files for profiling the defense systems. The reference *K. pneumoniae* (HS11286) was downloaded from the NCBI database.

**Identification of the defense systems, plasmids and resistance genes**

Prokaryotic Antiviral Defense Locator (PADLOC) was used for profiling of defense systems. The genes encoding the defense systems were identified by matching protein sequences with curated database (Hidden Markov Models-HMM) with >700 families of defense systems related proteins. DefenseFinder and KEGG were used for confirmation. The PADLOC output was loaded into Geneious prime for visualization of CRISPR-Cas and R-M systems. TADB3.0 was used to predict different types of TA pairs. Existing sequences of experimentally validated TA-proteins were also inputted into TBLASTN and PSI-BLAST programs. Searches were performed against the annotated MDR *K. pneumoniae* genomes and plasmids with E-value >0.05. Mobile genetic element finder (MGE-Finder v1.0.3) was employed for identifying plasmids (and other MGE). Comprehensive Antibiotic Resistance Database (CARD) and Resistance finder (ResFinder) for resistance genes.
